# Systemic Expression of Notch Ligand Delta-Like 4 during Mycobacterial Infection Alters the T Cell Immune Response

**DOI:** 10.3389/fimmu.2016.00527

**Published:** 2016-11-24

**Authors:** Matthew A. Schaller, Ronald M. Allen, Soichiro Kimura, Cheryl L. Day, Steven L. Kunkel

**Affiliations:** ^1^Department of Pathology, University of Michigan Medical School, Ann Arbor, MI, USA; ^2^Department of Microbiology and Infectious Diseases, Toho University School of Medicine, Tokyo, Japan; ^3^Department of Microbiology and Immunology, Emory University School of Medicine, Atlanta, GA, USA; ^4^Emory Vaccine Center, Emory University School of Medicine, Atlanta, GA, USA; ^5^South African Tuberculosis Vaccine Initiative (SATVI), Institute of Infectious Diseases and Molecular Medicine, School of Child and Adolescent Health, University of Cape Town, Observatory, South Africa

**Keywords:** Notch, hematopoiesis, monocytes, tuberculosis

## Abstract

The Notch ligand delta-like 4 (DLL4) is known to fine-tune the CD4^+^ T cell cytokine response. DLL4 is expressed on the surface of antigen-presenting cells (APCs) in a MyD88-dependent manner. We found that DLL4 expression was upregulated on bone marrow progenitor cells and APCs in mice infected with BCG *Mycobacterium*. Transfer of DLL4^+^ progenitor cells from infected hosts resulted in an increase DLL4^+^ myeloid cells in the spleen, indicating that expression of the *dll4* gene is propagated throughout hematopoiesis. We also found an increase in DLL4^+^ monocytes from individuals who were infected with *Mycobacterium tuberculosis*. In latent individuals, DLL4 expression correlated with increased cytokine production from T cells in response to PPD stimulation. Finally, antibody blockade of DLL4 reduced T cell cytokine production from naïve T cells stimulated with antigen. These results demonstrate that the Notch ligand DLL4 can influence T cell cytokine production in both humans and mice, and further reveal that expression of DLL4 is upregulated on early hematopoietic progenitors in response to chronic mycobacterial infection. These data suggest that widespread DLL4 expression may occur as a result of mycobacterial infection, and that this expression may alter CD4^+^ T cell responses to both previously encountered and novel antigens.

## Introduction

There is a growing body of evidence that the innate immune response to pathogen exposure can extend beyond the site of infection to alter both the rate of production and the function of newly formed white blood cells. For example, hematopoietic stem cells are known to respond to IFNγ with increased cell cycling (1–3), and bone marrow progenitor cells are known to respond to TLR agonists both *in vitro* and *in vivo* (4–7). Some of these changes may result in alterations in the innate immune response, a concept known as trained immunity (8, 9). In one study, trained immunity resulting from vaccination with bacille Calmette–Guerin was correlated with decreased morbidity resulting from pathogen exposure, and this vaccination resulted in overall reduced mortality compared to unvaccinated individuals (10).

Although many of these studies focus on altered differentiation of innate immune cells, there is little evidence that changes that occur at the stem cell level as a result of pathogen exposure can also alter the adaptive immune response. If changes relevant to adaptive immunity did occur in myeloid progenitor cells, these alterations would be most relevant in the adaptive immune response to chronic infection. Chronic infections are the result of immune responses that sub-optimally contain a pathogen at the expense of the host, resulting in tissue damage.

*Mycobacterium tuberculosis* (mTB) is responsible for approximately 1.5 million deaths each year and has infected between one-quarter and one-third of the world’s population (11–13), thus classifying this disease as a worldwide epidemic. The immune response to mTB is characterized by the formation of granulomas comprised of necrotic tissue and a milieu of cells that work in concert to contain the bacterium without effectively clearing it. Many researchers have demonstrated the importance of T cell participation in the containment of mTB (14–17). A recent study further suggests that the CD4^+^ T cell:antigen-presenting cell (APC) interaction plays a critical role in pathogen clearance and requires direct contact between the APC and the CD4^+^ T cell for an effective immune response (18). Other works have identified that APC-expressed costimulatory molecules aid in proper T cell activation in response to mycobacterial antigens (19, 20).

In the studies outlined below, we suggest that the Notch system provides an ideal target for long-term regulation of the adaptive immune system by APC in the setting of mTB infection. The Notch system consists of five ligands that signal promiscuously through four receptors to activate target gene transcription (21). The receptors Notch 1 and Notch 2 are expressed at every stage of the T cell lifecycle, and Notch signaling has demonstrated importance in thymic maturation (22, 23), effector function (24, 25), and the formation and maintenance of immunological memory (26). The Notch ligand delta-like 4 (DLL4), which aids in T cell differentiation, is specifically upregulated on APCs only as a result of TLR signaling *via* a MyD88 pathway (27). Several studies have demonstrated the importance of DLL4 in the T cell response in multiple diseases including respiratory syncytial virus (28), experimental autoimmune encephalomyelitis (29), type 1 diabetes (30), and the mycobacterial-elicited pulmonary immune response (31). DLL4 expression by APCs during viral infection reduced IL-4 and IL-13 production and increased production of IFNγ (28). In the mycobacterial- and EAE-driven responses, DLL4 increased IL-17 production from CD4^+^ T cells (29, 31, 32). In type 1 diabetes, blockade of DLL4 decreased T cell activation and increased T regulatory cell differentiation (30). These data suggest that the DLL4 ligand can alter the T cell-driven immune response in a context-specific manner.

Here, we outline a pathway whereby DLL4, which is upregulated in the lung during BCG mycobacteria infection in mice, is also increased systemically on the cell surface of APC as a result of exposure to this pathogen. Increased DLL4 protein can be observed on early progenitor cells in the bone marrow and on APCs in the spleen. We also demonstrate that DLL4 expression is maintained during short-term hematopoiesis in the absence of pathogenic stimuli using bone marrow chimeras. Finally, we demonstrate that DLL4 is upregulated on peripheral blood monocytes in a cohort of patients with latent or active TB infection and demonstrate that the presence of DLL4 on monocytes correlates with the T cell immune response in those patients that are latent tuberculosis infection (LTBI).

## Materials and Methods

### Mycobacterial Culture

BCG *Mycobacterium* (TICE strain) was obtained from Merck. Initial CFU was approximated by weight and cultures started in 3.5 mL 7H9 media supplemented with OADC (BD Biosciences). Tubes were kept in a 37°C incubator and agitated daily for 17 days. Cultures were spun at 3000 × *g* for 10 min and the supernatants removed. Pellets were resuspended in 3 mL of PBS with 0.5% Tween 80. Frozen stocks were prepared by mixing 20% of the BCG culture with 20% glycerol, 10% OADC, and 50% 7H9 media. Cultures were stored at −80°C. CFU was determined by serial dilutions (100–1,000,000×) of frozen stocks and plating on 7H11 media supplemented with 10% OADC.

To determine lung CFU, whole lungs were homogenized in 1 mL of PBS and serial dilutions from 10 to 10,000× plated on 7H11 agar supplemented with 10% OADC. Plates were incubated at 37°C for 16 days.

#### Mice

We used female C57BL/6 mice purchased from Taconic that were 6–8 weeks old at the beginning of the experiment. Mice were infected with 1.0 × 10^5^–5.0 × 10^5^ live *Mycobacterium* (BCG strain) by non-surgical intratracheal instillation.

#### Histology

Lungs were dissected and inflated with 10% normal buffered formalin and fixed in the same buffer overnight. Samples were then stored in 70% ethanol until processed. Histological analysis was performed on two serial 6-μm sections hematoxylin and eosin stained sections from each mouse.

#### RNA Extraction

RNA was extracted from 1.0 × 10^7^ splenic cells using TRIzol (Thermo Fisher Scientific) and 500 ng total RNA reverse transcribed using iScript (Bio-Rad, Hercules, CA, USA). QPCR was run on a Taqman 7500 analyzer using SYBR Green master mix and specific primers for murine *dll4* (Forward: AGGTGCCACTTCGGTTACACAG, Reverse: CAATCACACACTCGTTCCTCTCTTC). *Gapdh* was used as a control gene.

### Flow Cytometry

#### Murine

Flow cytometry was done on an LSR II with 488, 633, and 405 nM lasers. All mouse antibodies were purchased from BioLegend and used at a 1:200 dilution to stain between 1.0 × 10^4^ and 1.0 × 10^7^ cells in 200 μL of FACS buffer (containing 1% FCS and 0.002 M EDTA). In order to analyze only viable cells in the lung, the LIVE/DEAD Fixable Violet Dead Cell Stain Kit (Life Technologies) was used according to manufacturer’s instructions. All lung samples were analyzed by first gating on viable cells and subsequently determining populations of interest. For lung flow cytometry, minced lungs were digested in 5 mL of a 1 mg/mL solution of Collagenase A (Roche) and 10 Kunitz units of DNAseI (Sigma-Aldrich) in complete medium for 45 min at 37°C in a shaking incubator. Samples were subsequently passed 15× through a 5-mL syringe with an 18 G needle, and filtered through 100 μM Nitex (Wild Life Supply Co., Yulee, FL, USA) to remove debris. The lung homogenate was centrifuged at 400 × *g* for 5 min, and the resulting cell pellet was resuspended in 2 mL of FACS buffer. 200 μL of this suspension was used for flow cytometry staining in a 96-well plate. Spleen flow cytometry was performed by mincing the spleen and pressing through a 40 μM cell filter (BD) with the plunger from a 3-mL syringe and rinsing with FACS buffer. Spleen cells were centrifuged at 400 × *g* for 5 min and the resulting cell pellet resuspended in 3 mL of FACS buffer. For flow cytometry using bone marrow cells, one femur and one tibia were flushed with flow buffer using a 27-G needle, and cells were pressed through a cell filter, as described above. Cells were centrifuged and resuspended in 1 mL of FACS buffer and 200 μL was used for staining. To stain mouse cells, 1 μL of FC block and all specific antibodies were added simultaneously and incubated for 10 min at room temperature on an orbital shaker. Plates were centrifuged at 400 × *g* for 5 min, the supernatant aspirated, and an additional 200 μL of FACS buffer added to wash the cells. After this wash step, the supernatant was aspirated and cells resuspended in 200 μL of formalin using a multichannel pipette. Cells were incubated for 10 min on an orbital shaker and then washed 2× in PBS, as described above. To determine cell counts, 10 μL of CountBright counting beads (Life Technologies) were added prior to running the samples on a flow cytometer. Cell counts were determined per manufacturer instructions.

For bone marrow chimera experiments, femurs, tibias, humerus, sacrum, lumbar, and thoracic vertebrae were dissected from each mouse and crushed using an autoclaved mortar and pestle in the presence of FACS buffer. Crushed bones were rinsed several times and subjected to additional crushing until no red matter was visible in the pestle. Cells were centrifuged as described and red blood cells lysed using AKC lysis buffer. The pellet was washed 3× with FACS buffer, resuspended in the remaining volume and then a biotinylated lineage depletion cocktail (FC block, anti-CD3, anti-CD19, anti-Nk1.1, anti-Ter119, and anti-GR1) was added, using 2 μL of each antibody per mouse. Cells were incubated for 10 min at 4°C and then anti-biotin beads (50 μL per mouse) (Miltenyi) were added. Cells were incubated for an additional 15 min at 4°C and then washed with 20 mL of FACS buffer. Cells were then passed through a CS column (Miltenyi) per manufacturer instructions (cells from three mice were pooled for one CS column). Flow through was centrifuged, the supernatant aspirated, and cells resuspended in the remaining volume of about 300 μL, and then stained with antibodies against c-kit APC, sca-1 Pe-Cy7, and also streptavidin FITC. LSK cells were sorted on a FACS Aria II with 488, 633, and 405 nM lasers.

#### Human

Participants for these studies were recruited from the Cape Town Area in South Africa and were all >18 years of age and sero-negative for HIV (33). LTBI was defined as a T cell cytokine response to ESAT-6 and/or CFP-10 with no previous history of TB disease or treatment. Diagnosis of active disease was based on symptoms, patient history, and sputum microscopy and/or culture. All patients with active disease were positive either by sputum-smear microscopy and/or *M. tuberculosis* growth in liquid culture of sputum. Cryopreserved PBMCs were thawed in a 37°C water bath and immediately added to 20 mL of warm RPMI. Cells were spun at 400 × *g* and resuspended in 1 mL of FACS buffer for human cells (PBS + 1% human serum albumin). About 200 μL of cells were used for analysis. Cells were stained with 1 μL CD14 Alexa700 (clone M5E2), 1 μL Dll4 PE (clone MHD4-46), 1 μL CD11b Pacific Blue (clone ICRF44), and 1 μL of a 1-mg/mL solution of Human IgG to block FC receptors. Cells were stained for 20 min at room temperature and the same procedure followed, as described above.

#### Bone Marrow Chimeras

Six-week-old C57Bl/6 mice were irradiated twice with 500 G 3 h apart on a rotating platform in a cesium irradiator. Cells were injected intravenously in 100 μL RPMI 3 h after the second irradiation.

### Cell Culture

Naïve T cells were isolated from OT-II transgenic mice (The Jackson Laboratory, Bar Harbor, ME, USA) using Naïve Cell Isolation Kit II from Miltenyi. APCs from BCG infected and naïve mice were obtained by making a single-cell suspension from the pooled spleens of three mice. Red blood cells were lysed using AKC buffer, and cells were washed 2× in FACS buffer. Cells were then resuspended in 500 μL FACS buffer and incubated with 50 μL of an antibody cocktail containing equal proportions of anti-CD16/32 and biotinylated antibodies to CD3, B220, and Ter119 (all antibodies from Biolegend). Cells were then washed, incubated with magnetic beads, and run through a CS column, as described above, for bone marrow chimera FACS. The flow through from the column was centrifuged, resuspended in 300 μL FACS buffer, and stained with antibodies to CD11c and CD11b. Cells were then washed, resuspended in 500 μL FACS buffer, and CD11c^+^CD11b^+^ cells sorted by FACS using a FACS Aria II cell sorter. OT-II T cells were cultured at 1.0 × 10^5^/100 μL in a 96-well flat bottom plate with 1.0 × 10^4^ FACS sorted DCs in complete RPMI [10% FCS (Atlas Biologicals, Fort Collins, CO, USA) 1% non-essential amino acids, 1% sodium pyruvate, 1% HEPES, 1% penicillin/streptomycin, and 1% l-glutamine] (media and all supplements from Lonza, Walkersville, MD, USA) for 72 h. Cell culture supernatants were harvested and analyzed using Bio-plex 200 instrument and reagents (Bio-Rad). Anti-Dll4 antibody and control Ig for cell culture were purified from the serum of immunized rabbits using a protein A column (Bio-Rad). This antibody has previously been verified to be specific for the Notch ligand DLL4 (28).

### Statistical Analysis

**Figure 1:**
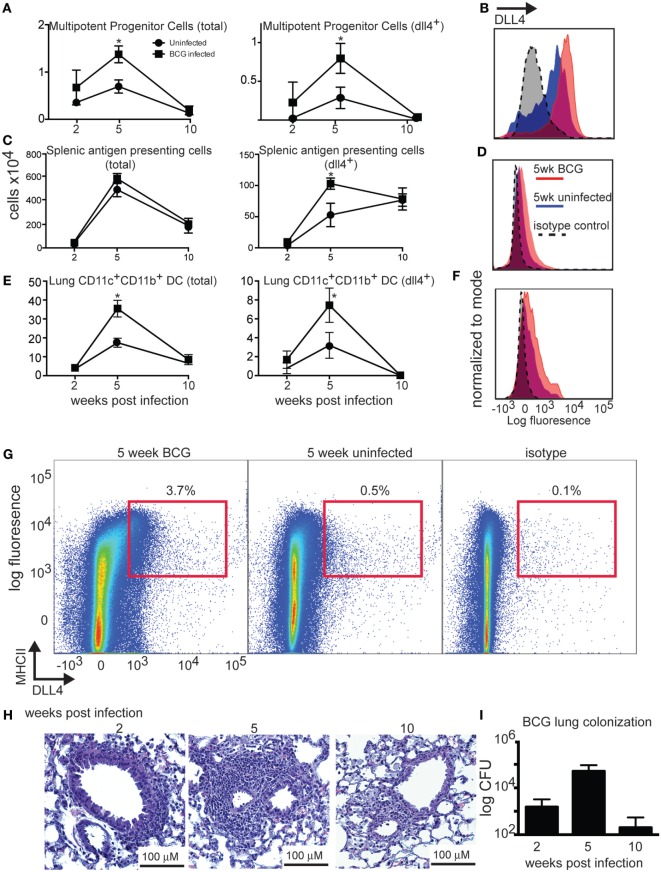
**Increase in number of cells expressing delta-like 4 in mice infected with *Mycobacterium***. **(A,C,E)** Increase in total and dll4^+^ multipotent progenitor cells (lin^−^ckit^+^sca1^+^flt3^+^), dll4^+^ splenic DC subsets (pooled together from CD4^+^CD11c^+^MHCII^+^, CD8a^+^CD11c^+^MHCII^+^, and CD4^−^CD8^−^B220^−^CD11b^+^CD11c^hi^MHCII^+^ populations), and dll4^+^ lung conventional dendritic cells (autoflourescent^−^CD11c^+^CD11b^+^MHCII^+^) at 5 weeks post-infection with 5.0 × 10^5^ BCG *Mycobacterium*. Two-way ANOVA indicated that time was a significant factor in our analysis (*p < 0.001). **(B,D,F)** Flow cytometry histograms indicating a slight but non-significant increase in DLL4 expression on the cell surface of MPP, B220^−^CD11c^+^MHCII^+^, and lung dendritic cells at 5 weeks post infection. **(G)** Flow cytometry dot plots depicting total spleen cells from a mouse infected for 5 weeks with BCG, an uninfected mouse from the same time point, and an isotype control. **(H)** Histology of murine lungs at 2, 5, and 10 weeks post-infection. **(I)** CFU counts from the lung at 2, 5, and 10 weeks post-infection. *N* = 5 mice per group, and the experiment was repeated three times.

Analysis was performed using Prism (all figures) or SPSS (all tables). In cases where ANOVA analysis indicated that one of the independent variables was a significant factor (see Figure 1), the cutoff for significance was adjusted to *p* < 0.001. Principle component analysis (PCA) was done using percent of DLL4^+^ monocytes and cytokine production (IL-2, IFNγ, or TNFα) from CD4^+^ T cells in response to specific antigens (Ag85A, CFP10, ESAT6, PPD, TB10.4, or *Staphylococcus* Enterotoxin B) from the same donor as individual variables (total of 19 variables per donor). After dimension reduction using direct oblimin rotation with Kaiser normalization, two components with an Eigenvalue ≥4 were identified in the cohort of latently infected individuals (Table 1). Component normalization indicated these components were not related (Table S3 in Supplementary Material).

**Table 1:** **Principle component analysis of cytokine production in response to multiple antigens including PPD, ESAT-6, TB4-10, CFP10, and AG85A, and DLL4 expression in patients with latent TB infection**.

	Component	
	Antigen specific	SEB stimulation
ESAT-6 (TNFα)	0.936	0.153
ESAT-6 (IFNγ)	0.925	0.086
PPD (IL-2)	0.875	−0.320
TB10.4 (IL-2)	0.866	0.042
PPD (TNFα)	0.861	−0.333
TB10.4 (IFNγ)	0.860	0.024
ESAT-6 (IL-2)	0.853	0.140
TB10.4 (TNFα)	0.848	0.046
PPD (IFNγ)	0.828	−0.371
Ag85A (IL-2)	0.590	−0.363
DLL4	0.561	−0.267
Ag85A (IFNγ)	0.469	−0.379
CFP-10 (IL-2)	−0.037	0.761
CFP-10 (TNFα)	−0.014	0.742
SEB (TNFα)	0.431	0.724
SEB (IFNγ)	0.498	0.723
SEB (IL-2)	0.593	0.718
CFP-10 (IFNγ)	−0.012	0.717
Ag85A (TNFα)	0.238	−0.306

### Patient Sample Collection and Animal Welfare

To acquire samples from human patients, this study was carried out in accordance with the recommendations of the Human Research Ethics Committee at the University of Cape Town and the Western Cape Department of Health with written informed consent from all subjects. The protocol was approved by the Human Research Ethics Committee at the University of Cape Town and the Western Cape Department of Health. These same de-identified samples were analyzed at the University of Michigan under IRB exemption HUM00065150, approved by IRBMED. Normal healthy donors were recruited (with written informed consent) from the University of Michigan using a protocol approved by the IRB committee (HUM00075841). All subjects gave written informed consent in accordance with the Declaration of Helsinki. For animal research, the experiments were carried out with recommendations from the Guide for the Care and Use of Laboratory Animals as written by the National Institutes of Health, University of Michigan Institutional Animal Care and Use Committee (IACUC). The protocol for animal use was approved by the University of Michigan IACUC (PRO00006469).

## Results

We have previously shown that DLL4 is upregulated in the lung in response to BCG infection in a TLR9-dependent manner (31). Experiments in mice have demonstrated that infectious processes at sites distal to the bone marrow can affect stem cell cycling and differentiation (1, 7). We hypothesized that mycobacterial infection may also alter the phenotype of bone marrow stem and progenitor cells. By investigating sites distal to the lung, we were able to determine that there is a systemic increase in the percent of cells expressing DLL4^+^ cells as a result of mycobacterial infection. In addition to the lung, we observed an increase in DLL4^+^ cells in spleen and bone marrow of infected mice, including all splenic DC subsets and bone marrow multipotent progenitors (Figures 1A,C,G). In the lung, an increase in DLL4 expression was most prominent on CD11b^+^ dendritic cells (Figures 1E,F), with no increase in DLL4 expression observed on alveolar macrophages or CD103^+^ DCs. Although we observed a slight increase in the mean fluorescence intensity of DLL4 as a result of infection, this did not reach statistical significance (Figures 1B,D,F). We observed that the increased DLL4 expression in the bone marrow and spleen was correlated with an increase in granuloma size and CFUs in the lung (Figures 1H,I). In the bone marrow, resolution of infection decreased the amount of DLL4 expressed on the surface of multipotent progenitor cells. We also observed age-related changes in DLL4 expression in uninfected mice, a finding that may reflect previous research demonstrating changes in Notch ligand expression in stromal cells as a result of time in human tissues (34). We were able to verify that our antibody was specific for DLL4 using dendritic cells derived from *Mx^Cre^Dll4^ff^* mice (Figure S1 in Supplementary Material). We did not observe an increase in expression of DLL4 in lymphocyte subsets (B cells, CD4^+^ T cells, and CD8^+^ T cells), hematopoietic stem cells, or common myeloid progenitor cells (Figure S2 in Supplementary Material). DLL4 + erythroid progenitors (pre-erythrocyte, pro-erythrocyte, or pre-megakaryocyte) were present in very low numbers and difficult to distinguish from background staining.

**Figure 2:**
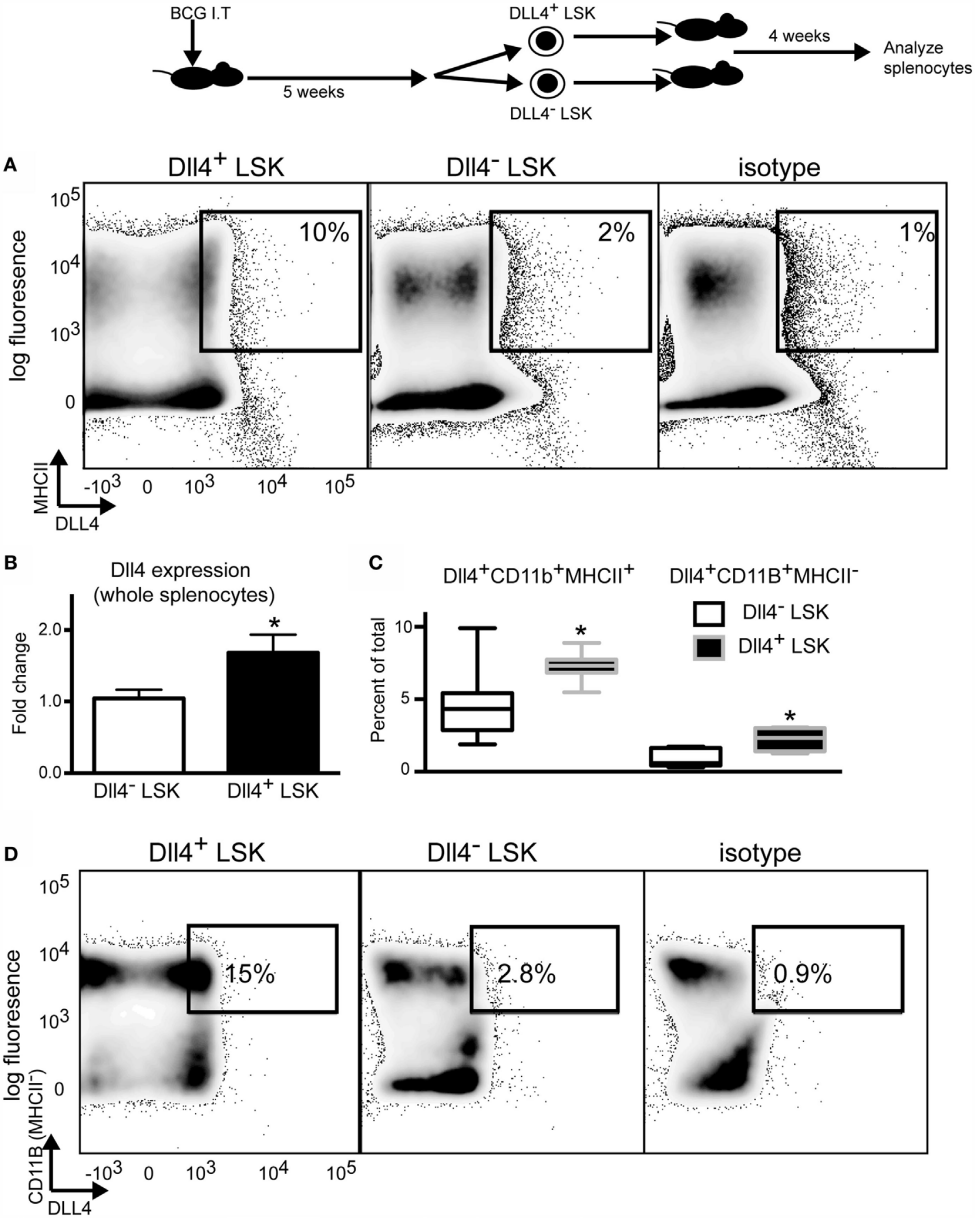
**Delta-like 4 expression is maintained during short-term hematopoiesis**. **(A)** Flow cytometry plots depicting dll4 and MHCII staining in total splenocytes from recipient chimera mice 4 weeks post-engraftment. Donor cells were either dll4^+^ LSK or dll4^−^ LSK cells isolated from mice infected with 5.0 × 10^5^ cells at 5 weeks post-infection. Each mouse received 5.0 × 10^4^ LSK cells. **(B)** QPCR analysis of whole splenocytes for *dll4* expression at 4 weeks post-engraftment. *N* = 4 mice per group, and the experiment was repeated two times. **(C)** Quantification of dll4 expression on B220^−^CD11B^+^MHCII^+^ and B220^−^CD11B^+^MHCII^−^ in the spleens of chimeric mice. *N* = 4 mice per group. **(D)** Flow plots depicting expression of dll4 on B220^−^CD11B^+^MHCII^−^ cells as quantified in **(C)**. Gates indicate percent of B220^−^CD11B^+^MHCII^−^ cells expressing DLL4. For **(B,C)**, Student’s *t*-test was performed (*p < 0.04). These experiments were repeated two times.

To determine if the expression of DLL4 was propagated throughout the hematopoietic system as a result of hematopoiesis, we isolated DLL4^+^ and DLL4^−^ Lineage^−^ Sca1^+^ cKit^+^ (LSK) cells from the bone marrow of BCG-infected mice by FACS. Isolated cells were engrafted into lethally irradiated hosts and splenocytes were assessed for DLL4 expression at several time points post engraftment. We observed a significant increase in DLL4 expression in the spleen by flow cytometry on CD11b^+^ MHCII^+^ cells isolated from recipient mice at 4 weeks post-engraftment (Figures 2A,B). QPCR from whole spleen demonstrated a 1.8-fold increase in expression of *dll4* at the RNA level (Figure 2B). We also observed an increase in DLL4 expression in CD11B^+^MHCII^−^ cells in the spleen; however, the majority of DLL4 expression was found in the MHCII^+^ population (Figures 2C,D). We did not observe an increase in expression of DLL4 on lymphocytes in these mice (Figure S3 in Supplementary Material). We did not observe an increase in DLL4 expression in mice receiving DLL4^+^ LSK cells at 7 days post-engraftment or at 8 weeks post-engraftment, suggesting that the expression of DLL4 as a result of hematopoiesis is transient in the absence of BCG infection, and is dependent on donor cell expression of this ligand. Using congenic mice, we verified that the majority of DLL4 expression was detected on donor cells and not on radio-resistant recipient cells at 4 weeks post-engraftment (Figure S3 in Supplementary Material).

**Figure 3:**
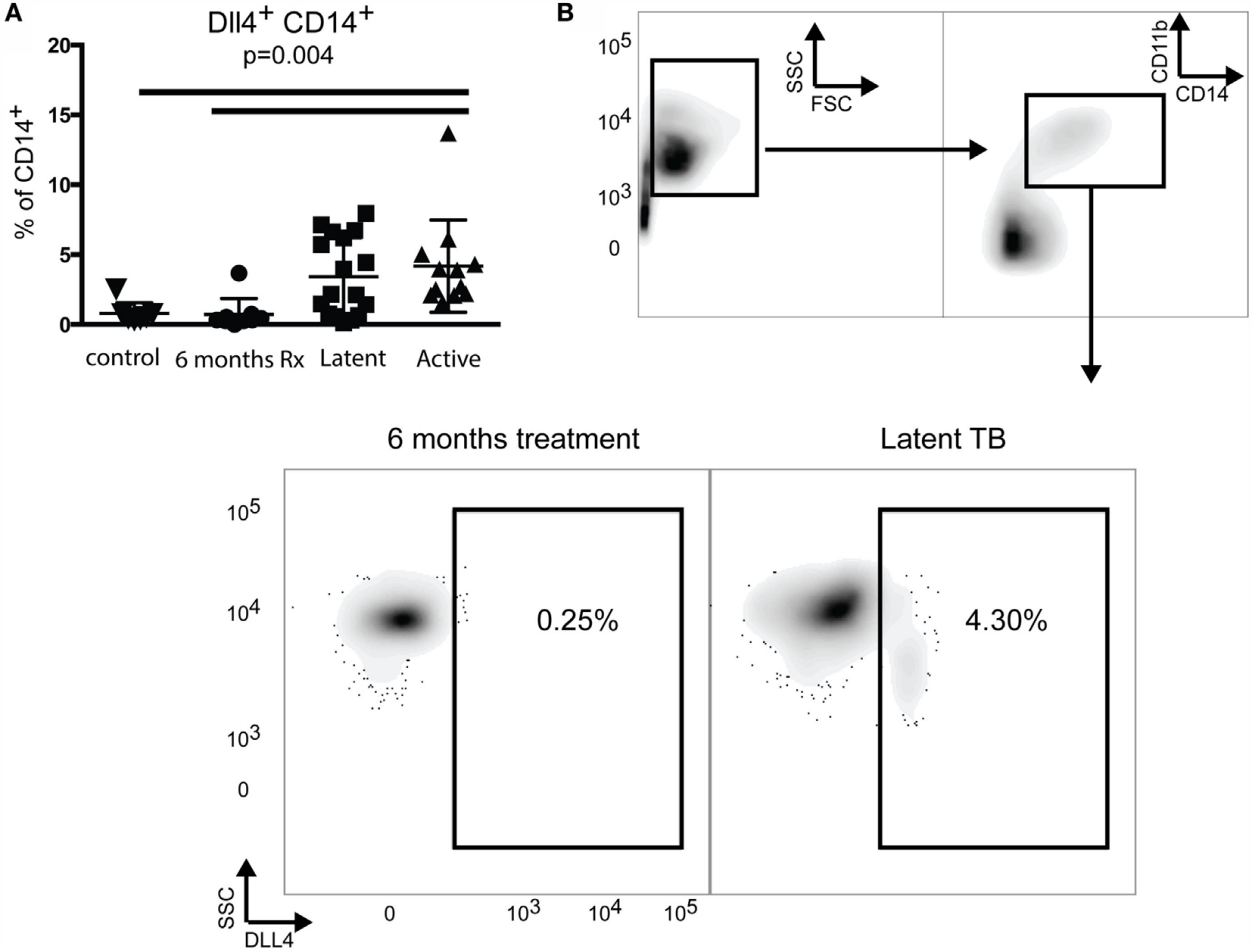
**Increased delta-like 4 expression in humans infected with Mtb**. **(A)** Analysis of expression of DLL4 on CD14^+^ monocytes in individuals diagnosed with latent or active *M. tuberculosis* infection. **(B)** Flow cytometry plots gated on CD14^+^ monocytes, depicting dll4 expression of an individual treated for 6 months compared to an individual latently infected with Mtb. One-way ANOVA analysis indicated that DLL4 was expression was significantly altered during Mtb infection (*p* = 0.0044).

We then hypothesized that expression of DLL4 could be detected on myeloid cells in the peripheral blood of humans with LTBI or active TB disease. As a negative control for these experiments, we used PPD negative healthy donors who were free of TB disease. Additionally, we tested those patients with active TB disease who had been successfully treated for 6 months with standard course anti-TB treatment. We found that those patients with LTBI or active TB disease had a significant increase in DLL4 expression when compared to both active TB patients following 6 months of treatment and normal healthy donors (Figure 3). When compared to normal healthy donors, this finding was statistically significant when we gated on CD14^hi^ PBMCs [*F*(3,41) = 3.207, *p* = 0.0044]. Comparison of expression of DLL4 on total PBMCs in our cohort of patients also resulted in a statistical significance as determined by ANOVA [*F*(3,36) = 1.776, *p* = 0.02], but no significant differences were found using Tukey’s multiple comparison test.

**Figure 4:**
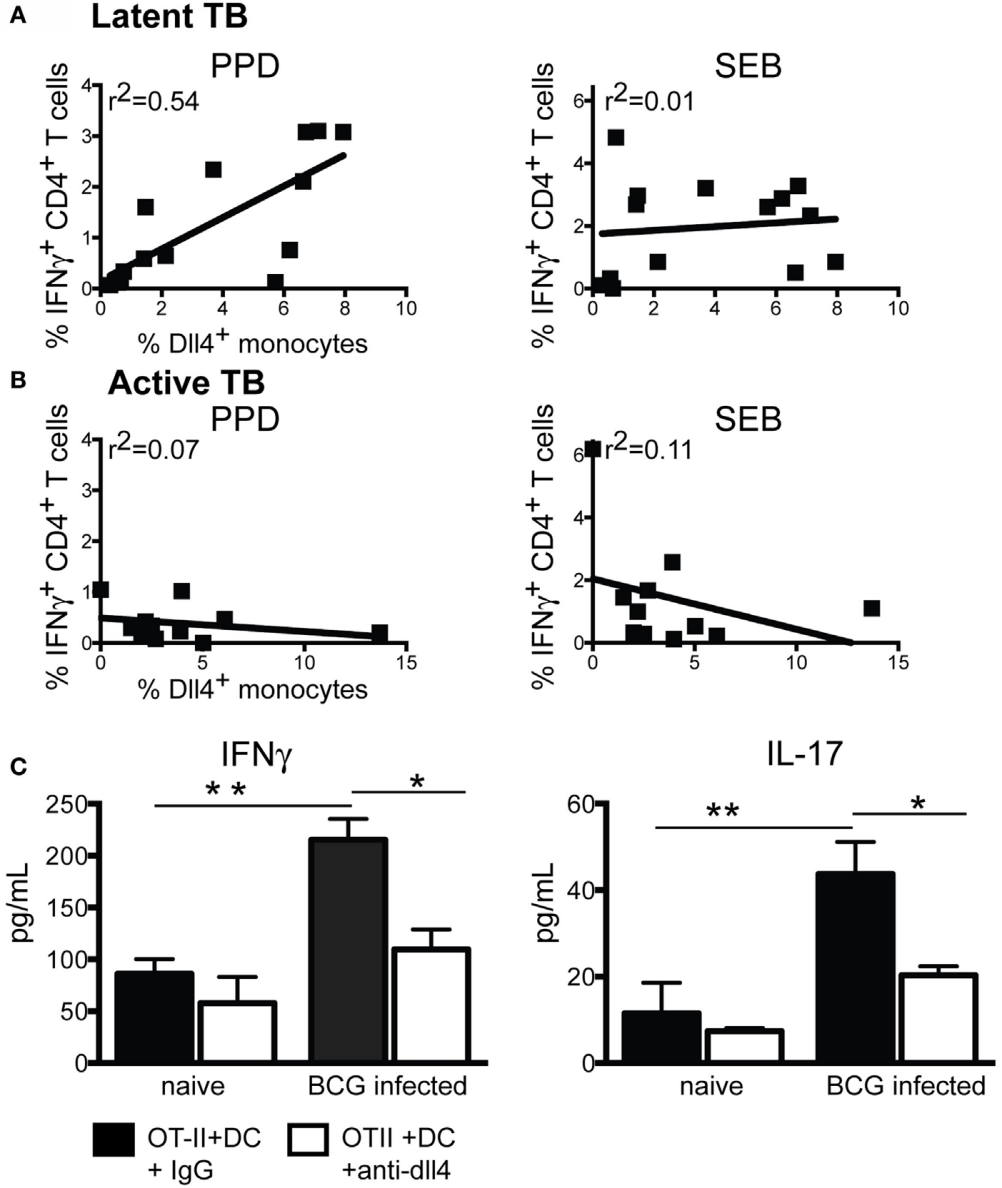
**Delta-like 4 expression correlates with cytokine production from T cells during latent infection**. **(A)** Linear regression analysis of CD4^+^ T cell IFNγ production, as measured by intracellular cytokine staining, compared to the percent of CD14^+^ monocytes expressing DLL4 in individuals diagnosed with latent Mtb infection. Similar results were obtained with IL-2 and TNFα production (Figure S4 in Supplementary Material). **(B)** Same comparison as **(A)** in individuals with active infection. **(C)** Production of IFNγ and IL-17 from murine OT-II cells cocultured with OVA peptide and splenic CD11c^+^CD11b^+^ FACS sorted from the spleens of naïve mice or mice infected for 5 weeks with BCG in the presence of a polyclonal antibody specific to DLL4 or IgG control antibody. Cells were cultured for 72 h in the presence of 10 μg/mL anti-DLL4 or control antibody. One-way ANOVA analysis indicated DLL4 was a significant factor in determining cytokine output from T cells. For IFNγ, the *p* value was <0.0001, and for IL-17 the *p* value was 0.02.

Notch ligand expression on APCs has been shown to alter the host immune response to several pathogens (35–39). For example, we have previously demonstrated that T cell-derived IL-17 production is increased as a result of Dll4 expression in a model of mycobacterial infection (31). To determine if DLL4 expression altered cytokine production from T cells in humans infected with *M. tuberculosis*, we assessed the CD4 T cell production of TNFα, IFNγ, and IL-2 in response to characterized mycobacterial antigens including ESAT-6, TB10.4, Ag85A, CFP-10, and PPD by intracellular cytokine staining in both actively and latently infected individuals. We also assessed the production of these same cytokines in response to the superantigen *Staphylococcal Enterotoxin B* (SEB) as a positive control. We then performed PCA using these data, including the percentage of DLL4^+^ monocytes as a factor to determine if DLL4 expression in monocytes was correlated with cytokine production from T cells in latently and actively infected individuals. PCA analysis is routinely used to reduce the number of dimensions in a data set by determining if specific variables track together as a component (40). Our analysis of latently infected individuals resulted in two components each with an Eigenvalue ≥4. The values in Table 1 state that the correlation between each variable and the two discovered principal components on a scale of 1 to −1, with 1 being a 100% correlation. Values of less than 0.3 are considered non-significant. Based on this analysis, we determined that DLL4 expression was correlated with cytokine production from CD4 T cells as a result of stimulation with specific TB antigens including ESAT-6 and PPD in an antigen-specific component. The second component contained all of the cytokine data related to SEB stimulation and was not correlated with DLL4 expression on monocytes. Interestingly, CFP-10 cytokine secretion was associated with the SEB component, suggesting a different pattern of cytokine secretion than that induced by the other antigens. Based on our component matrix (Table S1 in Supplementary Material), which correlates all variables in the data set, we determined that DLL4 expression was most closely correlated with the cytokine response following stimulation with PPD (Table S1 in Supplementary Material). Table S2 in Supplementary Material states that the component containing SEB cytokine stimulation was not related to the component containing DLL4 expression and cytokine production in response to TB antigens. Performing dimension reduction analysis on individuals diagnosed with active TB disease did not result in a component containing DLL4 expression. This analysis informed us that DLL4 expression on monocytes was associated with T cell cytokine production in latently infected individuals. We then performed linear regression analysis to determine if there was a direct correlation between cytokine production and DLL4 expression in both latent infection and active TB disease. Our results indicate an *r*^2^ value of 0.54 when correlating the percent of CD4^+^ T cells producing IFNγ and the percentage of monocytes expressing DLL4 in response to PPD. There was no correlation between DLL4 expression and cytokine production in response to SEB (Figure 4A). There was no correlation between cytokine production and DLL4 expression on monocytes in patients with active TB disease (Figure 4B). There was also no correlation between CD8^+^ T cell cytokine production and DLL4 expression (Table S3 in Supplementary Material). Consistent with previous data, there was also reduced frequency of cytokine-producing T cells in patients with active TB (33).

To experimentally verify that DLL4 expression on APCs that occurs as a result of BCG infection can alter T cell cytokine production from naïve T cells, we isolated dendritic cells (CD11c^hi^CD11b^+^) from the spleens of BCG infected mice and uninfected controls and cocultured them with T cells isolated from RAG2^−/−^ mice carrying the OT-II transgene. Cells were stimulated with the peptide OVA_323–339_ and in the presence of a blocking antibody to DLL4 or a control IgG. We observed that both IFNγ and IL-17 were increased in cocultures containing DCs from BCG-infected mice. This increase in cytokine production was reduced when a blocking antibody to DLL4 was added to the culture (Figure 4C).

## Discussion

Our data indicate that the number of cells expressing DLL4 is increased in both early hematopoietic progenitor cells and differentiated myeloid cells in the spleen as a result of lung mycobacterial infection in mice. This systemic upregulation is likely derived both from *de novo* exposure of immune cells to mycobacterial products and from the maintenance of *dll4* expression through hematopoiesis. As a small number of DLL4^+^ cells were derived from DLL4^−^ LSK cells (Figure S3 in Supplementary Material), we do not believe that exposure to *Mycobacterium* or mycobacterial products is the only way to induce expression of this ligand. Several studies support the notion that low-level DLL4 expression is a normal part of the hematopoietic process (41, 42). Our data suggest that the number of DLL4^+^ progenitor cells increases as a result of mycobacterial infection, and that this increased expression is passed on to the progeny of these cells, thus increasing the total amount of DLL4 found within the immune system. Therefore, it is likely that there are a small number of DLL4^+^ stem and progenitor cells that exist under non-infectious conditions, and these cells will generate DLL4^+^ daughter cells.

Our findings were confirmed using peripheral blood samples from a cohort of patients with either latent or active mTB infection. In both human and mouse, antigen-specific T cell cytokine production is influenced by DLL4 on the cell surface of APCs that occurs as a result of mycobacterial infection. It has previously been reported that certain T cell cytokine responses and expression of cell surface molecules including CD127 correlate with latent and active disease (33, 43–45). Although these changes in gene expression hint at a profound change in the innate immune system as a result of immune mTB infection, this response has not been fully characterized.

As the immune response to PPD is correlated with DLL4 expression in individuals with LTBI but not active TB infection, further study of the Notch system in TB may aid in characterization of the differences between immune responses in the setting of LTBI and active TB disease. Previous research suggests that DLL4 can alter the CD4^+^ T cell cytokine response and T cell survival (24–26, 32, 46, 47). Our current data indicate that DLL4 expression on APCs influences the T cell immune response during mTB infection. Furthermore, we demonstrate that DLL4 is upregulated on myeloid progenitor cells as a result of mycobacterial infection, and that this upregulated expression is passed on to daughter cells even after the microbial stimulus was removed. As we were able to observe a DLL4-dependent increase in OT-II T cell cytokine production when stimulating these cells with APCs from BCG-infected mice, we believe that the outcome of this aspect of trained immunity may extend beyond simple increased activation of the innate immune cells. The ability of DLL4 to increase the cytokine response of a naïve T cell, and the data we present here indicating that expression of this molecule is system wide, indicate that the entire immune response is “primed” for pathogen exposure.

It is of interest that DLL4 expression did not correlate with CD4 cytokine production in patients with active TB disease. This warrants further study and may indicate that Notch signaling is aberrant in those patients with active disease. One possibility is the increase in type 1 IFN pathway that is associated with active TB disease (48–51) may alter the expression of Notch ligands on the surface of myeloid cells. Type 1 IFN is known to increase in *dll1* expression in murine macrophages, and this ligand has been demonstrated to increase the T cell response to H1N1 influenza infection (35). In a model of T cell development, low levels of DLL1 and DLL4 expression were found to produce functionally distinct outcomes, with DLL4 inducing more expression of Notch target genes (52). Therefore, it is possible that the type 1 IFN pathway may alter the adaptive immune response to mycobacterial infection through altered Notch ligand expression. Further examination of monocytes from patients with active TB disease and individuals with LTBI may yield pertinent data on the role of Notch ligand expression in the anti-mycobacterial immune response.

It is of note that the ESAT-6-specific immune response has been documented as differing in both cytokine production and frequency of response in infected individuals when compared to the PPD-specific immune response (53). It is also possible that PPD, as a mix of antigens, elicits a stronger and more diverse response than a single antigen including ESAT-6 or others. These possibilities may explain why DLL4 expression correlates more strongly with one antigenic response than another.

The systemic wide upregulation of DLL4 may have effects on other cell types besides T cells. For example, macrophages are known to respond to Notch signaling with increased activation (54, 55). DLL4 is also essential for proper thymocyte development from common lymphoid progenitors (56). Therefore, a systemic increase in expression of this molecule may alter the host immune response in numerous ways that allow for more effective immune responses to pathogen challenge. Finally, as DLL4 expression is reduced in individuals who have been treated for mycobacterial infection, it is possible that the measurement of this ligand in peripheral blood will generate a biomarker to distinguish infected and uninfected individuals.

## Author Contributions

MS designed and performed experiments, and analyzed data. RA and SK performed experiments and analyzed data; CD designed and performed experiments, and analyzed data; SLK analyzed data and designed experiments.

## Conflict of Interest Statement

The authors declare that the research was conducted in the absence of any commercial or financial relationships that could be construed as a potential conflict of interest. The reviewers AF and CL and the handling editor declared their shared affiliation, and the handling editor states that the process nevertheless met the standards of a fair and objective review.
