# Supplementary material for: Systemic Expression of Notch Ligand Delta-Like 4 during Mycobacterial Infection Alters the T Cell Immune Response
Source: Front Immunol. 2016 Nov 24;7:527. doi: 10.3389/fimmu.2016.00527 (PMC5121470; doi:10.3389/fimmu.2016.00527)
Supplement: Supplementary file 1 [file Presentation_1.PDF]

Figure S1

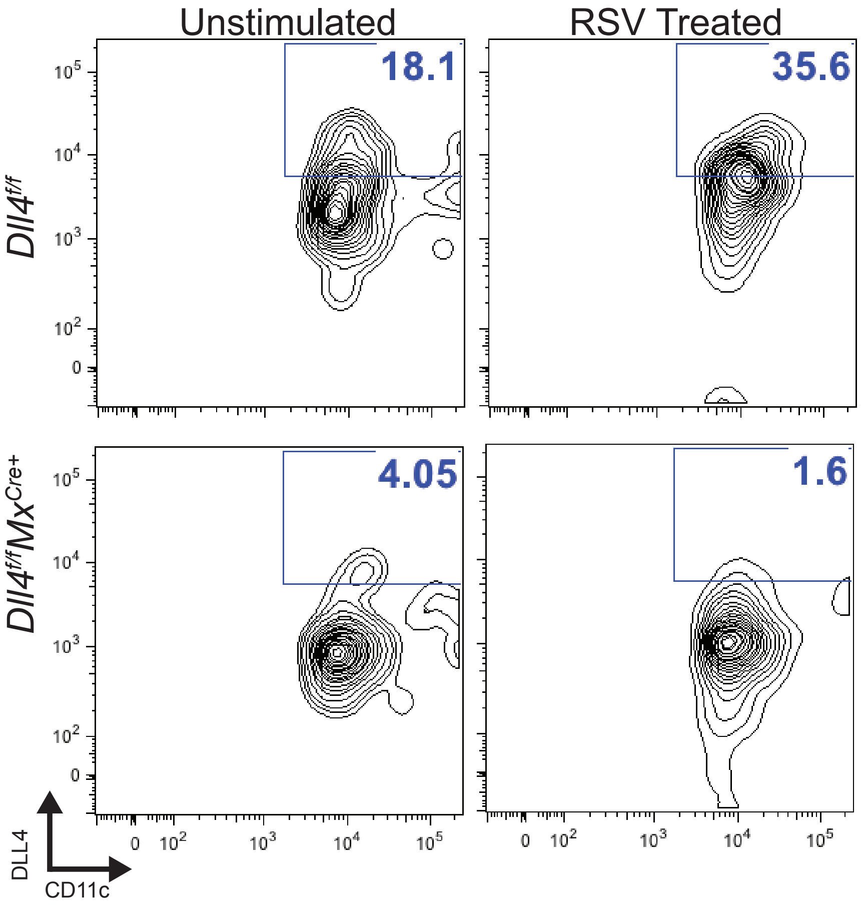

Unstimulated
RSV Treated
Dll4f/f
Dll4f/fMxCre+
18.1
35.6
4.05
1.6
DLL4
CD11c

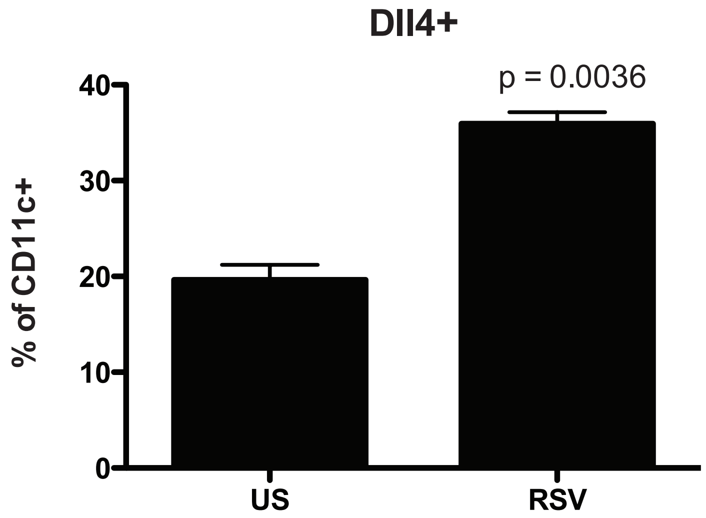

Dll4+
p = 0.0036
% of CD11c+
40
30
20
10
0
US
RSV

Figure S2

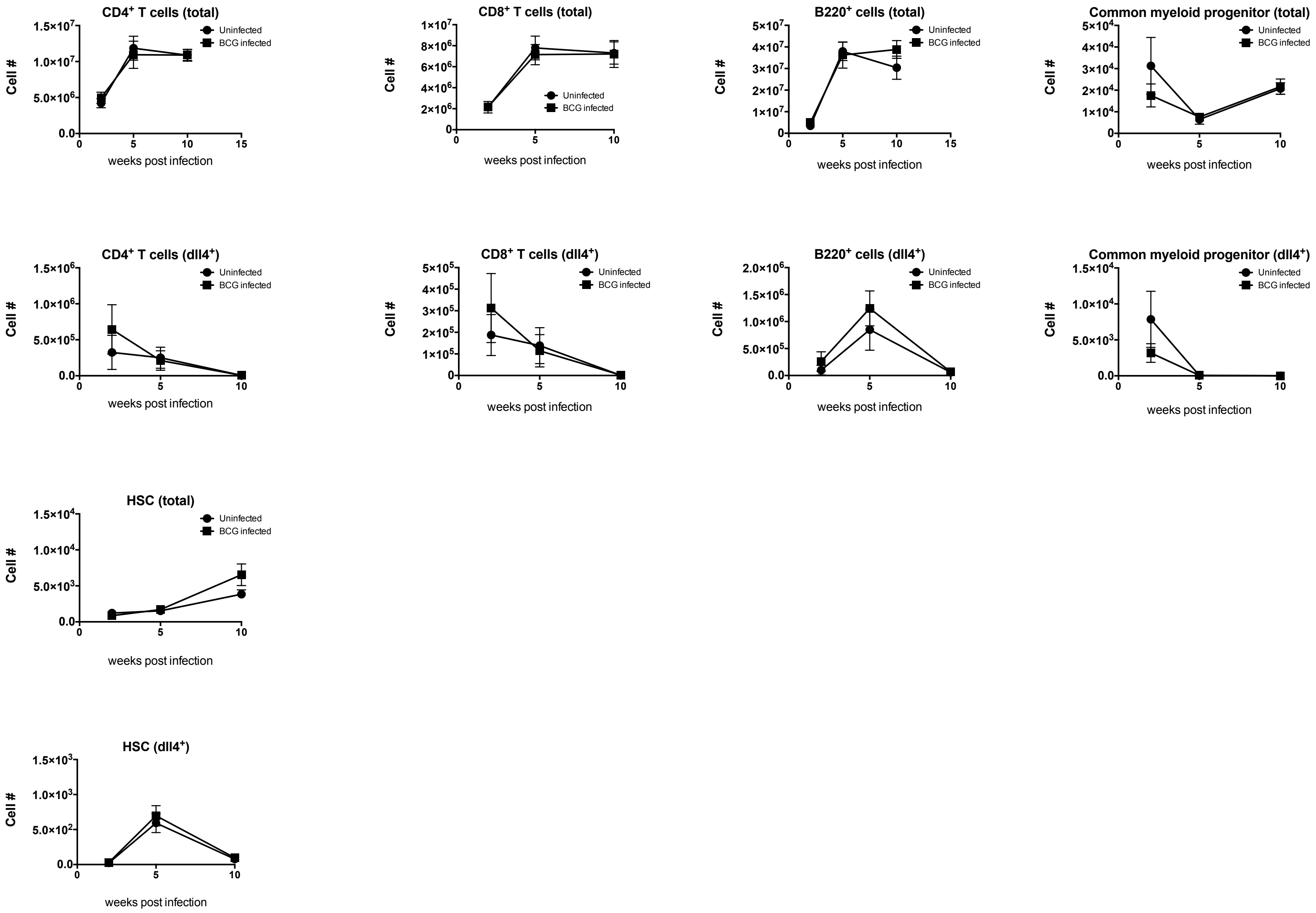
CD4+ T cells (total)
Uninfected
BCG infected
Cell #
1.5×107
1.0×107
5.0×106
0.0
0
5
10
15
weeks post infection
CD8+ T cells (total)
1×107
8×106
6×106
4×106
2×106
0
weeks post infection
B220+ cells (total)
5×107
4×107
3×107
2×107
1×107
Common myeloid progenitor (total)
5×104
4×104
3×104
2×104
1×104
CD4+ T cells (dll4+)
1.5×106
1.0×106
5.0×105
CD8+ T cells (dll4+)
5×105
4×105
3×105
2×105
1×105
B220+ cells (dll4+)
2.0×106
1.5×106
1.0×106
5.0×105
Common myeloid progenitor (dll4+)
1.5×104
1.0×104
5.0×103
HSC (total)
HSC (dll4+)
1.5×103
1.0×103
5.0×102

Figure S3

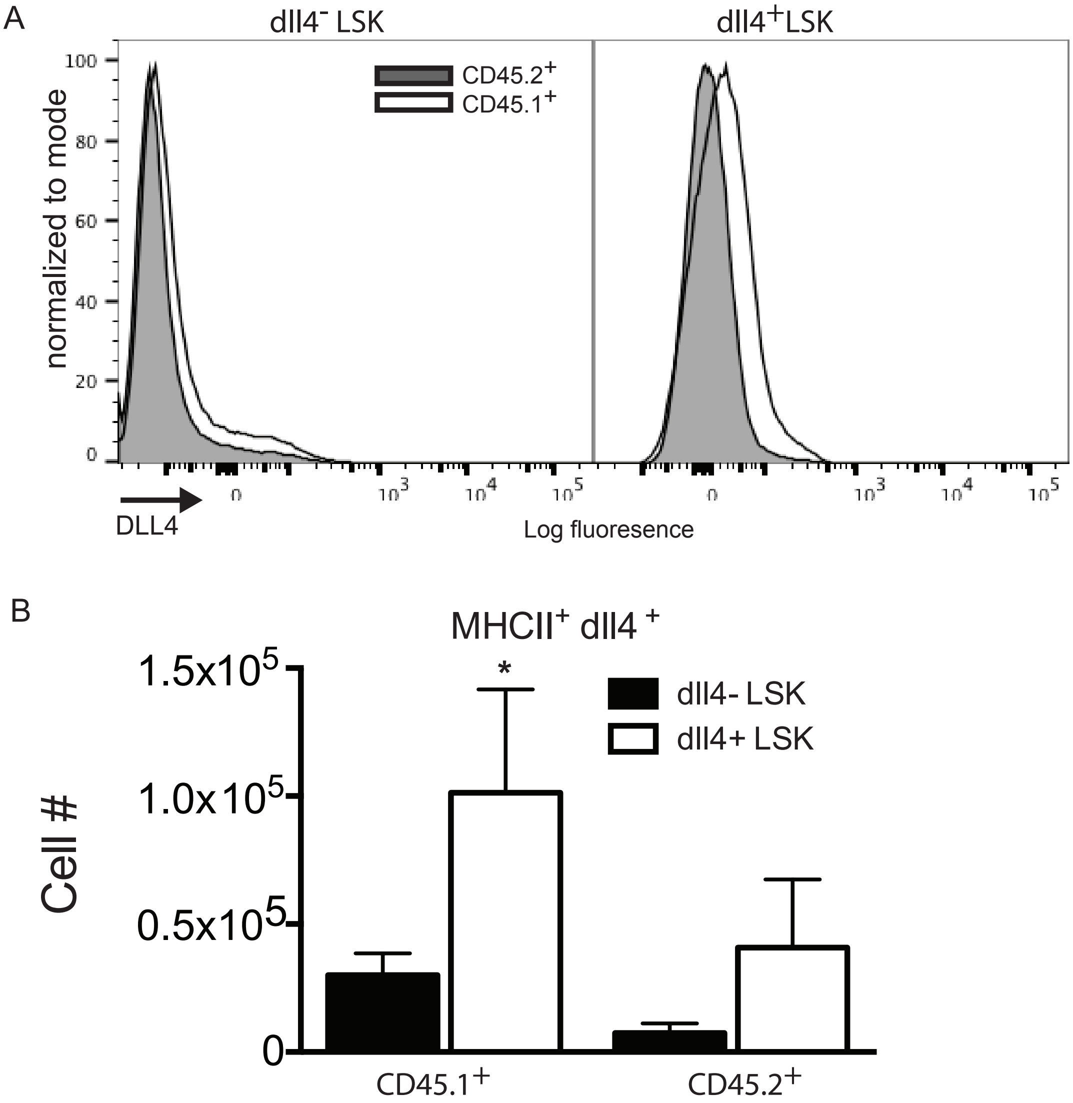

A
dll4⁻ LSK
dll4⁺LSK
CD45.2+
CD45.1+
normalized to mode
100
80
60
40
20
0
0
10^3
10^4
10^5
DLL4
Log fluoresence
B
MHCII+ dll4 +
*
dll4- LSK
dll4+ LSK
Cell #
1.5x10^5
1.0x10^5
0.5x10^5
0
CD45.1+
CD45.2+

Table S1: Correlation of CD4 T cell Cytokine Response and Dll4 expression on monocytes[a]

		dll4	Ag85A (IL-2)	CFP-10 (IL-2)	ESAT-6 (IL-2)	PPD (IL-2)	SEB (IL-2)	TB10.4 (IL-2)	Ag85A (IFNγ)	CFP-10 (IFNγ)	ESAT-6 (IFNγ)	PPD (IFNγ)	SEB (IFNγ)	TB10.4 (IFNγ)	Ag85A (TNFα)	CFP-10 (TNFα)	ESAT-6 (TNFα)	PPD (TNFα)	SEB (TNFα)	TB10.4 (TNFα)
Correlation	dll4	1.000	.288	-.123	.430	.676	.110	.304	.341	-.103	.501	.698	.099	.318	-.016	-.119	.461	.673	.016	.296
	Ag85AIL2	.288	1.000	.025	.451	.680	.009	.348	.877	.075	.501	.628	-.021	.338	.801	.059	.462	.630	-.158	.307
	CFP10IL2	-.123	.025	1.000	.155	-.131	.338	-.214	.016	.986	.103	-.178	.359	-.255	.035	.992	.101	-.170	.260	-.259
	ESAT6IL2	.430	.451	.155	1.000	.704	.492	.761	.195	.144	.972	.590	.335	.688	.089	.146	.962	.627	.309	.674
	PPDIL2	.676	.680	-.131	.704	1.000	.234	.620	.639	-.092	.791	.974	.190	.591	.286	-.105	.756	.978	.079	.565
	SEBIL2	.110	.009	.338	.492	.234	1.000	.626	-.061	.311	.513	.181	.964	.628	-.136	.337	.598	.232	.934	.642
	TB104IL2	.304	.348	-.214	.761	.620	.626	1.000	.105	-.223	.780	.567	.460	.975	.009	-.215	.823	.608	.448	.966
	Ag85AIFNg	.341	.877	.016	.195	.639	-.061	.105	1.000	.111	.325	.648	.020	.161	.805	.085	.289	.641	-.113	.142
	CFP10IFNg	-.103	.075	.986	.144	-.092	.311	-.223	.111	1.000	.116	-.125	.345	-.250	.138	.998	.113	-.118	.241	-.256
	ESAT6IFNg	.501	.501	.103	.972	.791	.513	.780	.325	.116	1.000	.703	.380	.745	.170	.116	.993	.741	.349	.735
	PPDIFNg	.698	.628	-.178	.590	.974	.181	.567	.648	-.125	.703	1.000	.158	.568	.278	-.142	.668	.997	.046	.543
	SEBIFNg	.099	-.021	.359	.335	.190	.964	.460	.020	.345	.380	.158	1.000	.483	-.113	.368	.466	.204	.963	.502
	TB104IFNg	.318	.338	-.255	.688	.591	.628	.975	.161	-.250	.745	.568	.483	1.000	.059	-.243	.794	.612	.483	.998
	Ag85ATNF	-.016	.801	.035	.089	.286	-.136	.009	.805	.138	.170	.278	-.113	.059	1.000	.115	.149	.280	-.195	.041
	CFP10TNF	-.119	.059	.992	.146	-.105	.337	-.215	.085	.998	.116	-.142	.368	-.243	.115	1.000	.115	-.133	.265	-.247
	ESAT6TNF	.461	.462	.101	.962	.756	.598	.823	.289	.113	.993	.668	.466	.794	.149	.115	1.000	.711	.442	.787
	PPDTNF	.673	.630	-.170	.627	.978	.232	.608	.641	-.118	.741	.997	.204	.612	.280	-.133	.711	1.000	.102	.590
	SEBTNF	.016	-.158	.260	.309	.079	.934	.448	-.113	.241	.349	.046	.963	.483	-.195	.265	.442	.102	1.000	.516
	TB104TNF	.296	.307	-.259	.674	.565	.642	.966	.142	-.256	.735	.543	.502	.998	.041	-.247	.787	.590	.516	1.000

a. This matrix is not positive definite.

Table S2: Component Correlation Matrix

Component	1	2
1	1.000	.009
2	.009	1.000

Extraction Method: Principal Component

Table S3: Correlation of CD8 T cell Cytokine Response and Dll4 expression on monocytes

		dll4	CD8 Ag85A (IL2)	CD8 CFP10 (IL2)	CD8 ESAT6 (IL2)	CD8 PPD (IL2)	CD8 SEB (IL2)	CD8 TB10-4 (IL2)	CD8 Ag85A (IFNg)	CD8 CFP10 (IFNg)	CD8 ESAT6 (IFNg)	CD8 PPD (IFNg)	CD8 SEB (IFNg)	CD8 TB10-4 (IFNg)	CD8 Ag85A (TNF)	CD8 CFP10 (TNF)	CD8 ESAT6 (TNF)	CD8 PPD (TNF)	CD8 SEB (TNF)	CD8 TB10-4 (TNF)
Correlation	dll4	1	-0.279	-0.195	0.146	0.104	0.191	-0.47	0.101	0.077	0.358	-0.322	0.158	-0.193	0.03	0.216	0.406	-0.078	0.094	-0.033
	CD8Ag85AIL2	-0.279	1	0.511	0.206	0.402	0.345	0.231	0.56	0.581	-0.485	-0.036	0.426	-0.042	0.347	0.683	-0.314	0.043	0.519	-0.014
	CD8CFP10IL2	-0.195	0.511	1	0.388	0.579	-0.223	0.199	0.278	0.124	-0.201	0.272	-0.028	-0.239	-0.085	0.316	-0.19	-0.029	0.004	-0.062
	CD8ESAT6IL2	0.146	0.206	0.388	1	0.581	-0.15	-0.056	-0.22	-0.21	0.444	-0.343	-0.105	-0.28	-0.275	-0.107	0.336	-0.142	-0.118	-0.203
	CD8PPDIL2	0.104	0.402	0.579	0.581	1	0.071	0.194	0.143	0.102	0.124	-0.137	0.1	-0.235	-0.418	0.312	0.021	-0.137	0.12	-0.135
	CD8SEBIL2	0.191	0.345	-0.223	-0.15	0.071	1	-0.173	0.241	0.553	-0.124	-0.18	0.78	-0.072	0.062	0.537	-0.164	-0.144	0.772	-0.168
	CD8TB410IL2	-0.47	0.231	0.199	-0.056	0.194	-0.173	1	-0.096	-0.047	-0.062	-0.007	-0.279	0.19	0.123	-0.211	-0.428	-0.294	-0.273	-0.187
	CD8Ag85AIFNg	0.101	0.56	0.278	-0.22	0.143	0.241	-0.096	1	0.429	-0.414	0.039	0.276	-0.202	0.545	0.79	-0.136	0.12	0.34	0.037
	CD8CFP10IFNg	0.077	0.581	0.124	-0.21	0.102	0.553	-0.047	0.429	1	-0.332	0.146	0.879	-0.033	0.144	0.824	-0.332	-0.113	0.888	-0.152
	CD8ESAT6IFNg	0.358	-0.485	-0.201	0.444	0.124	-0.124	-0.062	-0.414	-0.332	1	-0.077	-0.181	-0.057	-0.311	-0.332	0.665	-0.099	-0.23	-0.128
	CD8PPDIFNg	-0.322	-0.036	0.272	-0.343	-0.137	-0.18	-0.007	0.039	0.146	-0.077	1	0.145	0.558	0.123	0.109	0.234	0.641	0.189	0.582
	CD8SEBIFNg	0.158	0.426	-0.028	-0.105	0.1	0.78	-0.279	0.276	0.879	-0.181	0.145	1	0.001	0.018	0.729	-0.137	-0.028	0.986	-0.1
	CD8TB410IFNg	-0.193	-0.042	-0.239	-0.28	-0.235	-0.072	0.19	-0.202	-0.033	-0.057	0.558	0.001	1	0.314	-0.144	0.376	0.741	0.059	0.836
	CD8Ag85Atnf	0.03	0.347	-0.085	-0.275	-0.418	0.062	0.123	0.545	0.144	-0.311	0.123	0.018	0.314	1	0.257	0.07	0.363	0.073	0.321
	CD8CFP10TNF	0.216	0.683	0.316	-0.107	0.312	0.537	-0.211	0.79	0.824	-0.332	0.109	0.729	-0.144	0.257	1	-0.128	0.07	0.78	0.003
	CD8ESAT6TNF	0.406	-0.314	-0.19	0.336	0.021	-0.164	-0.428	-0.136	-0.332	0.665	0.234	-0.137	0.376	0.07	-0.128	1	0.622	-0.105	0.565
	CD8PPDTNF	-0.078	0.043	-0.029	-0.142	-0.137	-0.144	-0.294	0.12	-0.113	-0.099	0.641	-0.028	0.741	0.363	0.07	0.622	1	0.077	0.959
	CD8SEBTNF	0.094	0.519	0.004	-0.118	0.12	0.772	-0.273	0.34	0.888	-0.23	0.189	0.986	0.059	0.073	0.78	-0.105	0.077	1	-0.003
	CD8TB410TNF	-0.033	-0.014	-0.062	-0.203	-0.135	-0.168	-0.187	0.037	-0.152	-0.128	0.582	-0.1	0.836	0.321	0.003	0.565	0.959	-0.003	1

Supporting figure information:

Figure S1: Anti-dll4 antibody is specific for DLL4. A) Expression of *dll4* on murine bone-marrow derived DCs grown from $Mx^{Cre}dll4^{ff}$ mice that had been previously depleted of *dll4* by in vivo injection of poly IC. Cells were stimulated for 48 hours with Respiratory Syncytial Virus to induce *dll4* expression. Student's T test was performed to determine significance.

Figure S2: Analysis of DLL4 expression on lymphocytes, hematopoietic stem cells and common myeloid progenitor cells during murine BCG infection. There were no significant differences observed in the total number or the number of $dll4^{+}$ cells for the indicated populations. Two-way ANOVA indicated time was a significant factor that influenced the number of cells in each subset. N=5 mice per groups, experiment was repeated 2x.

Figure S3: DLL4 expression in chimeric mice is derived predominantly from donor cells. A) Flow cytometry histograms of dll4 expression in residual $CD45.2^{+}$ recipient cells and $CD45.1^{+}$ donor cells at 4 weeks post engraftment in mice receiving either $DLL4^{+}$ LSK cells or $dll4^{-}$ LSK cells isolated from mice at 5 weeks post BCG infection. B) Quantitation of A. One-way ANOVA indicated that the injection of $DLL4^{+}$ $CD45.1^{+}$ LSK cells was a significant factor in determining Dll4 expression on $CD45.1^{+}$ splenocytes at 4 weeks post engraftment. $p=0.0016$

Figure S4: DLL4 expression on monocytes is correlated with IL-2 and TNFα production from T cells during PPD stimulation. A-B) Linear regression analysis to determine the correlation between cytokine production for IL-2 and TNFα and

expression of DLL4 on human monocytes. Cytokine production was determined by intracellular cytokine staining after stimulation with PPD, ESAT-6 and SEB.

Table S1: Correlation matrix of $CD4^+$ T cell cytokine production and DLL4 expression on $CD14^+$ monocytes.

Table S2: Component correlation matrix from PCA analysis demonstrating that the resulting components from Table 1 are not related to each other.

Table S3: Correlation matrix for $CD8^+$ T cell cytokine secretion and DLL4 expression on monocytes.
